# Pathways to primate hip function

**DOI:** 10.1098/rsos.211762

**Published:** 2022-07-13

**Authors:** Lucrecia K. Aguilar, Clint E. Collins, Carol V. Ward, Ashley S. Hammond

**Affiliations:** ^1^ Department of Organismic and Evolutionary Biology, Harvard University, Cambridge, MA 02138, USA; ^2^ Division of Anthropology, American Museum of Natural History, New York, NY 10024, USA; ^3^ Department of Biological Sciences, California State University – Sacramento, Sacramento, CA 95819, USA; ^4^ Department of Pathology and Anatomical Sciences, University of Missouri, Columbia, MO 65212, USA; ^5^ New York Consortium of Evolutionary Primatology (NYCEP), New York, NY 10024, USA

**Keywords:** morphological system, functional morphology, locomotion, path analysis, structural equation modelling, primate evolution

## Abstract

Understanding how diverse locomotor repertoires evolved in anthropoid primates is key to reconstructing the clade's evolution. Locomotor behaviour is often inferred from proximal femur morphology, yet the relationship of femoral variation to locomotor diversity is poorly understood. Extant acrobatic primates have greater ranges of hip joint mobility—particularly abduction—than those using more stereotyped locomotion, but how bony morphologies of the femur and pelvis interact to produce different locomotor abilities is unknown. We conducted hypothesis-driven path analyses via regularized structural equation modelling (SEM) to determine which morphological traits are the strongest predictors of hip abduction in anthropoid primates. Seven femoral morphological traits and two hip abduction measures were obtained from 25 primate species, split into broad locomotor and taxonomic groups. Through variable selection and fit testing techniques, insignificant predictors were removed to create the most parsimonious final models. Some morphological predictors, such as femur shaft length and neck-shaft angle, were important across models. Different trait combinations best predicted hip abduction by locomotor or taxonomic group, demonstrating group-specific linkages among morphology, mobility and behaviour. Our study illustrates the strength of SEM for identifying biologically important relationships between morphology and performance, which will have future applications for palaeobiological and biomechanical studies.

## 1. Introduction

**Figure 1.**
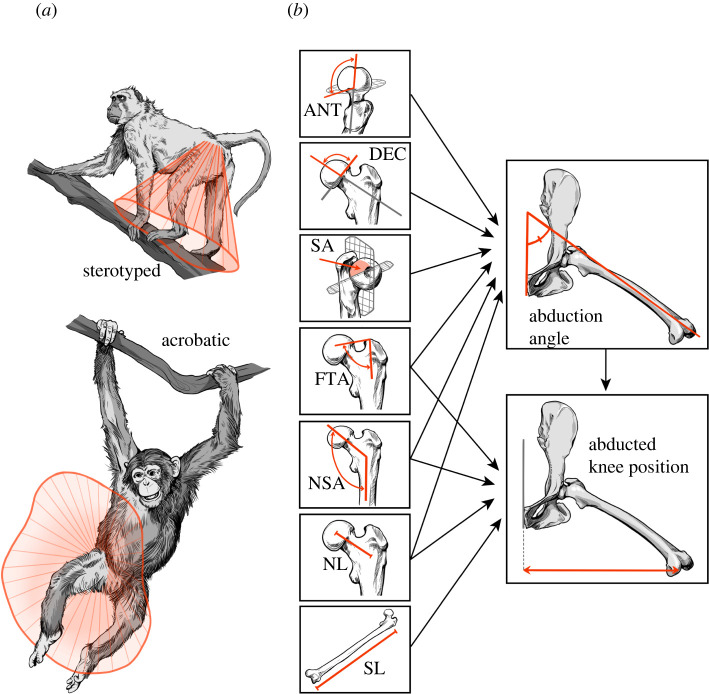
(*a*) The grasping envelope of the hindlimb (red outline) should differ between stereotyped and acrobatic primates. (*b*) Diagram of baseline recursive path model depicting relationships between morphological trait and hip abduction variables. *Femoral head anteversion* (*ANT*) and *declination* (*DEC*), *fovea-trochanteric angle* (*FTA*), *neck-shaft angle* (*NSA*) and *abduction angle* were all measured in degrees. *Femoral* *neck length* (*NL*), *shaft length* (*SL*) and *abducted knee position* were measured in millimetres. *Femoral head relative* *surface area* (*SA*) is a dimensionless ratio of posterosuperior subchondral bone area relative to the total surface subchondral bone of the femoral head.

A shift from primarily above-branch quadrupedal locomotion to a repertoire that includes below-branch positional behaviours represents a key transition in the evolution of extant hominoids [1]. A critical limitation for biomechanical and palaeontological studies is that the relationships between morphological traits and their resultant locomotor capabilities are difficult to quantify. Acrobatic behaviours (i.e. below-branch behaviours associated with arboreal versatility, including forelimb-dominated suspension, brachiation and vertical climbing) in hominoids require reaching distantly spaced discontinuous arboreal supports with the hands and feet (figure 1*a*). In theory, the reach of an animal in three dimensions—the grasping envelope (figure 1*a*)—should be increased by morphological adaptations that enhance mobility and length of the reaching limb [2–6]. This appears to be substantiated by data showing that apes and semi-suspensory prehensile-tailed ateline monkeys have a larger hindlimb envelope (vis-à-vis distance spanned at the knee during hip abduction) [2,3,7], although data for the forelimb envelope are less clear [8]. Multiple studies (e.g. [9–15]) have associated below-branch behaviours in apes and atelines with morphological features assumed to promote high levels of hip and shoulder mobility, especially range of limb abduction. However, non-human primate experimental studies that have yielded data on hip joint mobility (e.g. [2,7,16,17]) or kinematics (e.g. [18–21]) have not quantified bony anatomy in the same individuals.

Although a direct linkage between functional abilities and specific morphological traits of the primate hip has never truly been established, a hypothetical morphofunctional framework does exist. Morphologies interpreted as enhancing hip abduction are largely based on theoretical constraints of how the joint moves and positions during normal loading (e.g. [13,22–26]). Bony features of the pelvis and femur, soft tissues of the hip, and body size determine how far an animal can position their hindlimb, and thus dictate an animal's efficacy in the arboreal setting. Researchers have focused on morphological traits that hypothetically increase the range of positions of the femoral head within the acetabulum and/or allow for a greater joint excursion before impingement of soft or bony tissues. Features commonly hypothesized to increase hip joint mobility include a long and steeply inclined femoral neck, a short greater trochanter and a centrally positioned fovea capitis (when present) on a relatively large and spherical femoral head (e.g. [2,10,13,22,24–34]). Often overlooked, yet equally important for crossing gaps in a discontinuous arboreal environment, is that the reach of the hindlimb is also determined by factors unrelated to joint mobility, such as body size and limb segment lengths. Furthermore, determining which morphological traits are the most influential on hip joint mobility is crucial for predicting biomechanical performance.

A recent study [3] developed a method of digitally articulating primate femora and pelves from osteological specimens and generating simulations of hip abduction. The models of hip abduction were compared with *in vivo* hip abduction data from anaesthetized primates [3], with simulations showing close and consistent correspondence with the live animal data [2,7,35]. These hip abduction models provide the opportunity to examine hip function and morphological data for the *same individuals* in order to test how different traits are related within the same morphofunctional system. Here, we use structural equation modelling (SEM), a family of causal inference methods, to test hypothesized relationships among femur morphologies and hip mobility. We examine which bony morphological traits most strongly predict hip abduction in anthropoid primates using broadly different locomotor behaviours, as well as among major taxonomic groups. This study is the first of its kind to mathematically model anthropoid morphological traits and locomotor performance as a biological system.

## 2. Material and methods

### 2.1. Data collection

Seven femur morphological traits and two simulated hip abduction metrics were measured from osteological specimens representing 25 extant anthropoid species exhibiting diverse locomotor behaviours in the wild (table 1). Morphological traits were quantitatively characterized in PolyWorks v. 11.0 software (InnovMetric, Québec, Canada) from surface scans of bones. Measurements included *femoral neck length* (*NL*), *femoral shaft length* (*SL*), *neck-shaft angle* (*NSA*), *fovea-trochanteric angle* (*FTA*), *femoral head declination* (*DEC*), *femoral head anteversion* (*ANT*) and *femoral head relative surface area* (*SA*; a ratio of subchondral bone present on the posterosuperior femoral head relative to total SA). Detailed descriptions of measurement protocols are provided in electronic supplementary material S1 (text and figures S1–S4).

**Table 1.** Primate species included in analyses, with number of male and female specimens, taxonomic and locomotor groupings, and institution(s) at which specimens were accessed. Taxonomic group abbreviations: P, Anthropoid primate; H, Hominoid; M, Monkey. Locomotor group abbreviations: A, Acrobatic; S, Stereotyped. Note that three taxa (*A. caraya*, *P. nemaeus* and *G. beringei*) were excluded from locomotor analyses. Orangutans (*Pongo* spp.) were not included because adult orangutans lack a fovea capitis [36], which was integral to measurements used in this study. Institution abbreviations: AMNH, American Museum of Natural History, New York, NY, USA; CMNH, Cleveland Museum of Natural History, Cleveland, OH, USA; KNM, National Museums of Kenya, Nairobi, Kenya; MCZ, Museum of Comparative Zoology at Harvard, Cambridge, MA, USA; MRAC, Royal Museum for Central Africa, Tervuren, Belgium; USNM, United States National Museum, Washington, DC, USA; ZMA, Naturalis Leiden and Zoological Museum Amsterdam Collections, Leiden, The Netherlands; ZSM, Bavarian State Zoological Collections, Munich, Germany.

taxon	♂ (♀)	taxonomic groups	locomotor group	institution(s)
*platyrrhines (n = 33)*				
*Ateles belzebuth*	0 (2)	P, M	A	AMNH
*Ateles fusciceps*	2 (4)	P, M	A	USNM
*Ateles geoffroyi*	0 (3)	P, M	A	USNM
*Ateles paniscus*	3 (0)	P, M	A	ZMA, AMNH
*Alouatta caraya*	5 (5)	P, M	—	AMNH
*Cebus apella*	3 (6)	P, M	S	AMNH, USNM, ZMA
*colobines (n = 45)*				
*Colobus guereza*	2 (3)	P, M	S	USNM, KNM
*Nasalis larvatus*	8 (9)	P, M	S	MCZ, USNM, ZSM
*Procolobus badius*	0 (4)	P, M	S	PCM
*Rhinopithecus roxellana*	1 (2)	P, M	S	USNM, AMNH
*Trachypithecus cristatus*	8 (8)	P, M	S	MCZ, ZSM, USNM, ZMA
*cercopithecines (n = 62)*				
*Cercopithecus mitis*	4 (4)	P, M	S	USNM
*Erythrocebus patas*	3 (2)	P, M	S	AMNH, USNM, ZSM
*Macaca fascicularis*	4 (6)	P, M	S	MCZ, USNM
*Macaca nemestrina*	1 (5)	P, M	S	MCZ
*Papio cynocephalus*	1 (7)	P, M	S	KNM, USNM
*Papio anubis*	4 (5)	P, M	S	USNM
*Pygathrix nemaeus*	5 (2)	P, M	—	USNM, AMNH, MCZ
*Theropithecus gelada*	3 (6)	P, M	S	UZIA, USNM, ZMA
*hominoids (n = 91)*				
*Hylobates lar*	7 (10)	P, H	A	ZSM, MCZ
*Symphalangus syndactylus*	10 (11)	P, H	A	ZSM, USNM, ZMA, AMNH
*Gorilla gorilla*	10 (8)	P, H	A	CMNH, USNM
*Gorilla beringei*	3 (3)	P, H	—	USNM
*Pan troglodytes*	8 (10)	P, H	A	CMNH, USNM
*Pan paniscus*	5 (6)	P, H	A	MRAC

The two hip abduction measurements used in this study come from previously published reconstructions of primate hip abduction [3]. These models provide an ideal system in which to test structure–function linkages because the abduction (i.e. functional) data are derived from the same individuals as the morphological (i.e. structural) data. Our method for reconstructing and validating primate hip abduction has been described in detail elsewhere [3]. Briefly, laser scan data were collected for a single innominate and corresponding femur for each specimen. Microscribe landmark data were collected from the entire articulated pelvis, including median plane landmarks on the sacrum and pubic symphysis. Landmark data were imported into PolyWorks software and used to digitally register each innominate model to the three-dimensional landmarks, positioning the innominate relative to its median plane. Best-fit spheres were fit to the acetabular lunate surface and femoral head subchondral bone, with the femoral head sphere made coincident with the lunate surface sphere. The centroid of these two coincident spheres established the joint rotational pivot for all movements of the femur, allowing combinations of motions across all three rotational degrees of freedom. We did not allow for translational movements of the femur, as in some non-mammalian studies (e.g. [37,38]), because the ball-and-socket structure of the primate hip joint contains bony and soft tissue structures that limit translational movements *in vivo* (i.e. cup-like acetabulum [10,25], labrum [39–41] and capsular ligaments [42]).

The femur was then rotated into an abducted posture following four anatomically determined constraints. (i) The femur was not allowed to interpenetrate the pelvis. (ii) The borders of the femoral head and acetabulum were not allowed to overlap. (iii) The fovea capitis (ligamentum teres attachment) was not allowed to move beyond the acetabular fossa. The ligamentum teres is usually contained within the acetabular fossa [13,22,23], a positioning theorized to reduce cartilage microtrauma and joint degeneration from ligament impingement [43,44]. Moreover, the ligamentum teres restricts femoral movements in extreme joint positions [45–47], and the position of the fovea capitis may thus provide an indication of normal mammalian joint positions (e.g. [22]). (iv) The long axis of the femur was oriented orthogonal to the main axis of the ischium when abduction was measured. In other words, the femur was allowed to move through all three degrees of freedom as it was moved into an abducted position but measurements were taken with the long axis of the femur oriented orthogonal to the ischium for consistency in positioning among individuals. By incorporating these anatomical constraints on joint positioning, the reconstructions do not reflect the maximum possible joint postures; greater hip abduction would probably be possible in reconstructions considering all possible joint positions, including more flexed, rotated and/or translated postures.

*Abduction angle* was defined as the angle formed between the long axis of the femoral diaphysis and the midline plane. *Abducted knee position* was defined as the distance between a point set in the intercondylar fossa and midline plane, representing the farthest point from the midline that an animal's knee can reach in the abducted posture. This latter measurement represents a proxy for the hindlimb envelope.

All relevant data are included in electronic supplementary material S2.

### 2.2. Predictions

To identify important relationships between femoral morphological traits and hip abduction measurements in primate groups, we built a graphical framework of hypotheses for how these variables interact in order to specify a baseline recursive path model (figure 1*b*). *Neck-shaft angle* (*NSA*) was predicted to affect *abduction angle* because a larger angle positions the femoral head above the femoral neck, allowing more hip abduction before the neck and greater trochanter impinge on tissues around the joint. *NSA* was also predicted to determine *abducted knee position* because a larger angle positions the knee further laterally for any given position of the hip. Similarly, *fovea-trochanteric angle* (*FTA*) was predicted to determine both measures of abduction because the vertex of this angle tracks the height of the greater trochanter and overall geometry of the femur. *Neck length* (*NL*) was predicted to determine *abduction angle* because a longer femoral neck should allow more manoeuvrability around the hip joint. *NL* probably tracks body size to some extent, so this variable was also predicted to determine *abducted knee position*. *Shaft length* was predicted to determine only *abducted knee position*, as it has no apparent impact on manoeuvrability at the joint itself. Lastly, measures of femoral head articular surface distribution and orientation (i.e. *declination, anteversion)* influence the way the femoral head moves in the acetabulum and were thus predicted to only determine the *abduction angle*. *Abduction angle* was predicted to affect the *abducted knee position*.

Based on this framework, the baseline, theoretical path model was defined in *lavaan* [48] R code as

$$\begin{array}{rl} & \mathrm{Abduction}\;\mathrm{angle}∼ANT+DEC+SA+FTA+NL+NSA\\ & \mathrm{Abducted}\;\mathrm{knee}\;\mathrm{postition}∼FTA+SL+NL+NSA+\;\mathrm{abduction}\;\mathrm{angle} \end{array}$$

### 2.3. Analyses

Data were split into three taxonomic (Anthropoid, Hominoid and Monkey) and two locomotor (Acrobatic and Stereotyped) groups (table 1). The Anthropoid group (*n* = 231 individuals) included all specimens from the full dataset of anthropoid primates. The Hominoid group (*n* = 91 individuals) included all great and lesser ape specimens. The Monkey group (*n* = 140 individuals) included platyrrhine and cercopithecid monkeys, with the latter group comprising the majority.

Taxa were assigned to two broad locomotor groups (Acrobatic or Stereotyped) based on their reported usage of below-branch locomotor behaviours (electronic supplementary material S1 (text and table S1)). These locomotor classifications are considerable simplifications of the diverse behaviours exhibited by anthropoids, but are nonetheless necessary to test hypotheses regarding functional differences among taxa. For the purposes of this study, ‘Acrobatic’ taxa are those characterized by below-branch locomotor versatility that allows them to negotiate almost all arboreal substrate sizes, orientations and continuities with ease. The Acrobatic group (*n* = 99 individuals) included extant hominoids and the prehensile-tailed *Ateles* [49–59]. Taxa with little or no reported use of forelimb-dominant anti-pronograde behaviours (e.g. both arboreal and terrestrial quadrupeds) were assigned to the ‘Stereotyped’ group. ‘Stereotyped’ here does not refer to the measure of correlation strength between two or more motions [60], because the lack of carefully collected high-speed video in the wild prevents this level of analysis. Instead, Stereotyped is defined by the preference for quadrupedal, pronograde movements even when negotiating substrate gaps. We chose this classification based on natural history and ecological observations. The Stereotyped group (*n* = 109 individuals) included capuchins, cercopithecines and colobines [61–69]. *Pygathrix* [70,71], *Alouatta* [67,72,73] and *Gorilla beringei* [51,74] could not be easily classified as either acrobatic or stereotyped based on the published literature and were thus excluded from locomotor analyses (but do remain in the taxonomic group path models).

Linear measurements were natural log-transformed and angular measurements were converted to radians to ameliorate potential issues related to non-normality, scale and allometry. Body size was not directly assessed in this study even though it is a variable that probably affects all biological relationships, and these are some of the most well-studied relationships in biology [75–77]. We allowed body size to be directly integrated in our study while using log-transformed data because it is unlikely that measures of body mass could be reliably estimated in all of the osteological specimens, and because many body size correction techniques facilitate opaque or misleading interpretations in multi-variate statistical methods [75]. For each taxonomic and locomotor group, morphological traits and hip abduction measurements were tested for normality using quantile–quantile plots, density plots and Shapiro–Wilk tests. Correlation matrices were generated with Spearman's (*ρ*) rank and Pearson (*r*) correlation coefficients for each group. As multi-collinearity can cause disruptions in path analyses such as decreasing stability of model parameters [78,79], we calculated variance inflation factors (VIF)—a measure of inflation of the standard error between two variables—among all variables within each group. A high VIF can indicate overweighting of variable magnitudes in path models. We considered a VIF of greater than 10 unacceptable (following [78]). Normality testing and correlation matrices were conducted in R (v. 3.6.3, [80]); VIF calculations were performed in JMP^®^ Pro (v. 15.0.0, SAS Institute Inc).

We used SEM to conduct path analysis to test and improve upon the baseline path model of morphological trait and hip abduction variables (figure 1*b*) in each taxonomic and locomotor group [81]. This technique estimates *a priori* associations between multiple predictor and one or more response variables, allowing for the evaluation and modification of causal relationship hypotheses. Path analysis is a specific form of SEM that does not model latent (unmeasured) variables. Scaled data from each taxonomic and locomotor group were independently fit to the baseline path model using either standard (ML) or robust (MLR) maximum-likelihood estimation methods (see [82] for estimator choice reasoning). ML was used for stereotyped group models only, as all other groups violated the normality assumptions of ML, thus necessitating the use of MLR. We chose to consider non-independence due to shared ancestry only at the general level of taxonomic groupings because more granular phylogenetic SEM can obscure predictive power [83].

Regularization, a variable selection procedure used most commonly in linear regression, has recently been applied to SEM [84,85]. We implemented regularized SEM using the *lasso* penalty technique to determine significant predictor variables (i.e. morphological traits) from each taxonomic and locomotor group's baseline model. This allowed us to optimize explanatory power and parsimony by penalizing the addition or exclusion of factors that did not significantly improve model fit. Insignificant variables were trimmed, resulting in more parsimonious models. Data were then fit to these updated models. When applicable, we also created and tested further modified model options by removing variables with insignificant estimates (*p* > 0.05 and standardized estimate less than 0.3) and/or returning variables with high correlation residuals (greater than 0.1). Our method for refining models allowed us to find the most predictive and efficient model for each group based on sound theory and statistical procedure.

All candidate models (baseline and refined) for each group were assessed and compared to identify best-fit final models. Correlation residuals were analysed for local fit testing of individual causal paths (with less than or equal to 0.1 considered acceptable); model *χ*^2^ test statistic (*p* > 0.05 acceptable), root mean square error of approximation (RMSEA; less than or equal to 0.1 acceptable), comparative fit index (CFI; greater than or equal to 0.9 acceptable) and standardized root mean square residual (SRMR; less than or equal to 0.1 acceptable) were examined for global fit testing. Within each taxonomic and locomotor group, models were compared with fit indices (Akaike information criterion (AIC), expected cross-validation index (ECVI)). Note, however, that Kline ([81]; see Ch. 11–12) cautioned against over-reliance on specific fit index and comparison cut-offs. If any models were near-equivalents, the most parsimonious model was chosen as the final model.

All SEM analyses were conducted in R with packages *lavaan* [48], *regsem* [86] and *semPlot* [87]. Relevant code for all analyses is provided in the electronic supplementary material S3.

## 3. Results

In total, we produced and tested three path model iterations for the Anthropoid group, two for Hominoid, four for Monkey, three for Acrobatic and four for Stereotyped from our model refinement procedures (see electronic supplementary material S3). Though the baseline model for the Acrobatic group demonstrated suitable fit, baseline models for all other groups exhibited relatively poor fits. Within every group, refined final models demonstrated good local and global fits, and outperformed baseline models across nearly every metric. Model comparison testing showed lower or near-equivalent AIC and ECVI scores for final models versus those of other candidate models in each group. Fit and comparison statistics for baseline and final models from each group are given in table 2.

**Table 2.** Results of fit and comparison testing for SEM analyses of baseline and final path models fit with data from each of three taxonomic (Anthropoid, Hominoid, Monkey) and two locomotor (Acrobatic, Stereotyped) groups. Metrics include the χ^2^ test statistic (with degrees of freedom (d.f.) and sample size (*n*)), CFI, RMSEA (with 90% confidence interval (CI)), SRMR, AIC and ECVI. Relevant *p*-values are included.

group	model	$\chi^{2}$ (d.f., *n*)	CFI	RMSEA [90% CI]	SRMR	AIC	ECVI
Anthropoid^a^	baseline	9.776 (4, 231), *p* = 0.044	0.996	0.067 [0.000, 0.122]	0.005	349.582	0.143
	final	5.367 (4, 231), *p* = 0.252	0.998	0.046 [0.000, 0.134]	0.020	−948.168	0.094
Hominoid^a^	baseline	7.349 (4, 91), *p* = 0.119	0.991	0.101 [0.000, 0.214]	0.005	122.387	0.374
	final	4.064 (3, 91), *p* = 0.255	0.997	0.070 [0.000, 0.221]	0.006	−475.906	0.276
Monkey^a^	baseline	14.795 (4, 140), *p* = 0.005	0.985	0.105 [0.051, 0.164]	0.007	373.218	0.246
	final	1.898 (4, 140), *p* = 0.754	1.000	0.000 [0.000, 0.106]	0.019	−3.326	0.148
Acrobatic^a^	baseline	5.294 (4, 99), *p* = 0.258	0.997	0.062 [0.000, 0.185]	0.005	135.281	0.325
	final	0.620 (1, 99), *p* = 0.431	1.000	0.000 [0.000, 0.257]	0.007	−539.815	0.169
Stereotyped	baseline	9.746 (4, 109), *p* = 0.045	0.982	0.115 [0.016, 0.209]	0.010	323.848	0.328
	final	0.677 (2, 109), *p* = 0.713	1.000	0.000 [0.000, 0.138]	0.015	−443.827	0.135

^a^Due to MLR estimation method used, robust versions of *χ*^2^, CFI, RMSEA and SRMR are given.

**Figure 2.**
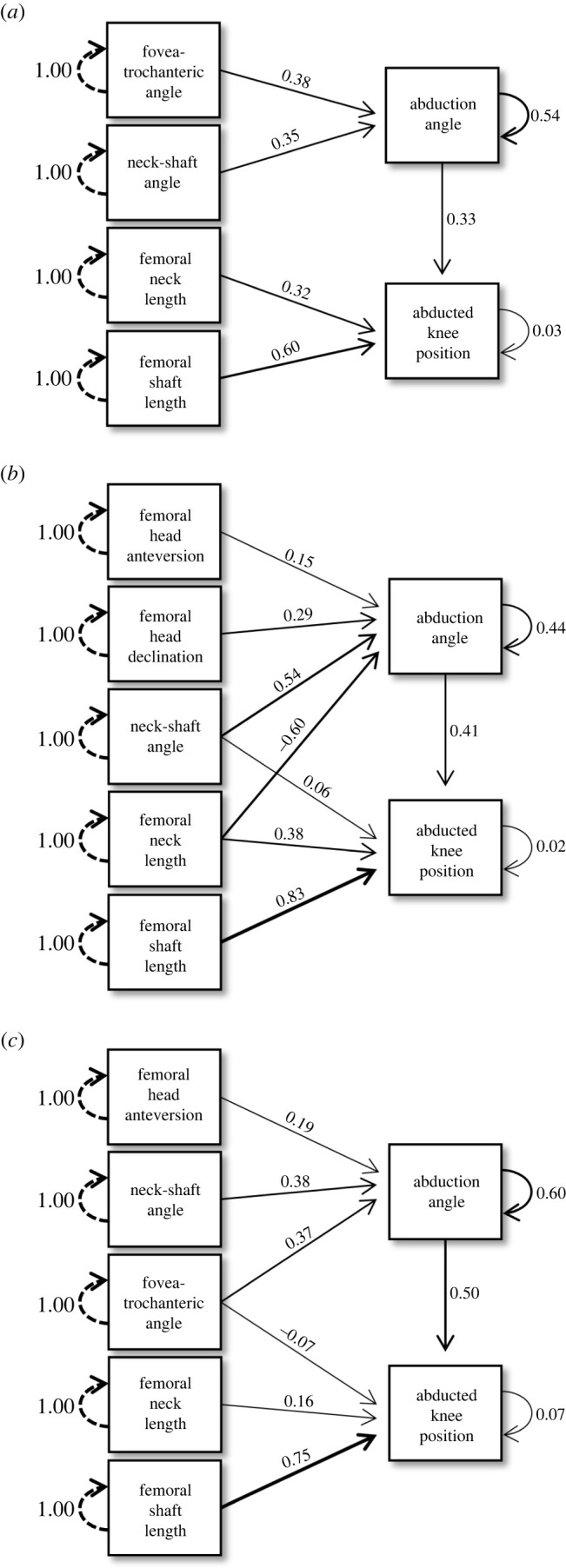
Diagrams of final path models depicting relationships between femur morphological traits and hip abduction measurements in the (*a*) Anthropoid group, (*b*) Hominoid group and (*c*) Monkey group. All path coefficients shown are standardized estimates.

**Figure 3.**
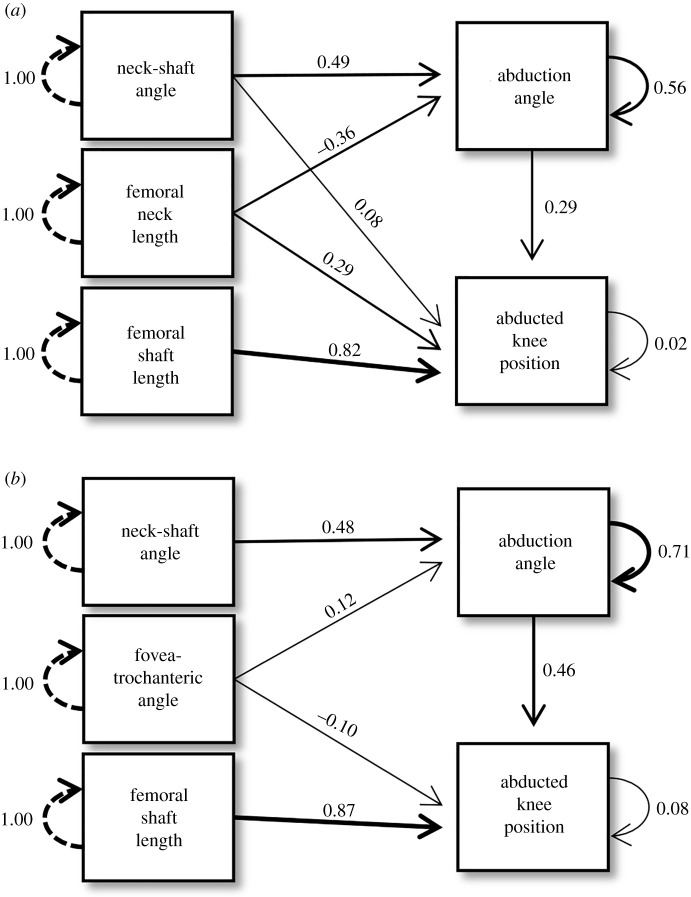
Diagrams of final path models depicting relationships between femur morphological traits and hip abduction measurements in the (*a*) Acrobatic group and (*b*) Stereotyped group. All path coefficients shown are standardized estimates.

Final model path diagrams for each taxonomic and locomotor group are shown in figures 2 and 3, respectively. Final models differed among groups, but some morphological variables proved important in multiple groups. *SL* was a strong predictor of *abducted knee position* across all groups; *NL* predicted *abducted knee position* in all groups except Stereotyped. *NSA* predicted *abduction angle* in every group. Additionally, *abduction angle* was a good predictor of *abducted knee position* across all groups, with standardized coefficient estimates ranging from 0.29 to 0.50. *SA* did not appear in any final models. All paths appearing in the Acrobatic group final model also appeared in the Hominoid model and all paths appearing in the Stereotyped group model appeared in the Monkey model, which was anticipated given overlap in sample compositions.

Correlation matrices and VIF are provided in the electronic supplementary material S1 (tables S2–S11). Following criteria outlined in the methods, we did not detect multi-collinearity issues that would unfairly weight any model parameters or compromise the integrity of our model interpretation.

## 4. Discussion

We employed SEM to test and quantify linkages between morphological traits of the femur and hip abduction abilities in anthropoid primates. These linkages were considered across broad taxonomic and locomotor groups. Our study demonstrates one of the first uses of regularized SEM for modelling a system combining morphological traits and performance data. SEM is a powerful tool for testing and visualizing hypothetical structure–function relationships due to the integrated nature of morphology and performance [88–90], as was clear in our results. Using regularized SEM allowed us to settle on models that accurately optimize both parsimony and explanatory power, thus elucidating core structure–function relationships in the hip. Although a few morphological traits (e.g. *NSA* and *SL*) stood out as being strong predictors of hip function generally, systematically developing path models also allowed us to distinguish among functional locomotor groups more specifically. By following SEM model modification guidelines, we were able to statistically distil the complex and integrated nature of hip abduction down to its essential osteological parts.

Two morphological traits are shown here to be broadly useful in predicting hip function in anthropoids: *NSA* and *SL*. Specifically, larger *NSA*s increase the angle of abduction of the thigh in primates and longer femoral shafts increase the distance spanned by the knees. Among all paths in our models, the strongest relationships were between *SL* and *abducted knee position*. Yet, surprisingly, the other metric that should track anthropoid body size (i.e. *NL*) shared a negative relationship with *abducted knee position* (discussed more below). There is some nuance in applying these results. As *NSA* confers information about *abduction angle* (and via *abduction angle*, provides indirect information about *abducted knee position*), it can provide some insight into arboreal versatility. Acrobatic behaviours cannot be inferred directly from *SL*, but a longer femoral shaft increases the hindlimb envelope and is thus complementary to acrobatic postures. All morphological traits were related to our measures of hip performance in one or more groups, except for *SA*. It is possible that this morphological trait simply does not reflect hip abduction in non-human anthropoids. Alternatively, our measurement (a ratio of subchondral bone on the posterosuperior aspect relative to the total subchondral surface) may not be sophisticated enough to detect differences in articular surface distribution between groups, with approaches such as three-dimensional geometric morphometrics needed to capture articular surface shape in the future.

There is substantial overlap in locomotor and taxonomic group compositions. The Stereotyped group is made up exclusively of monkeys, while the majority of species in the Acrobatic group are hominoids. These group make-ups allow us to further explore the results of both the taxonomic (Anthropoid, Hominoid and Monkey) and locomotor (Acrobatic and Stereotyped) path models. *DEC* provides an additional significant path to *abduction angle* in the Hominoid model compared with the Monkey model, suggesting that the femoral head orientation in apes enhances hip abduction as compared with the femoral head configuration of monkeys (even the acrobatic *Ateles*). The height of the greater trochanter, as tracked by *FTA*, was not significantly related to hip abduction in the Hominoid model, yet this morphological trait is favourable for hip abduction in the Monkey model. Interestingly, a comparison of the Monkey and Hominoid path models confirms that hip abduction is more than just a sum of its parts—distinct morphological traits are differentially important to each taxon.

One perplexing yet interesting result is the significant, negative relationship of *NL* to *abduction angle* in the Hominoid and Acrobatic models. In theory, a longer femoral neck should allow for more manoeuvrability of the femoral head within the acetabulum before the femur impinges on the bony pelvis and overlying soft tissues [3]. Not only does our result run counter to these expectations, but *NL* was also not negatively related to hip abduction in the other models. More research is needed to fully explain these findings, but interactions with other aspects of morphology or taxonomic composition of the samples may provide some initial explanation. First, a negative relationship between *NL* and other morphological variables in hominoids may exist. For example, *NL* and *NSA* have significant negative correlation coefficients in the Hominoid and Acrobatic groups, whereas the Monkey and Steretotyped groups show non-significant and/or positive correlations between these morphological variables (electronic supplementary material S1 (tables S2–S6)). There may be geometric changes associated with a higher *NSA* in the Hominoid and Acrobatic samples that result in a shorter femoral neck, possibly to minimize structural failure of the femoral neck while loaded in more diverse hindlimb postures. Second, *NL* may also track hominoid body size in a manner unaccounted for here. Gorillas (especially *G. beringei*) have the longest absolute *NL* among hominoids but display the most restricted hip abduction (electronic supplementary material S2), so gorillas may disproportionately influence the Hominoid path model results. The Acrobatic model, on the other hand, excludes *G. beringei* from the sample but includes smaller bodied *Ateles*. Although the inclusion of the small-bodied spider monkeys (and removal of large mountain gorillas) might be expected to reduce the negative trend between *NL* and *abduction angle*, the short *NL* coupled with large *abduction angle* in *Ateles* may actually strengthen the negative relationship.

Applications to anthropoid primates serve, paradoxically, as an impetus and a limitation in this study. Gathering both osteological and locomotor performance data *in vivo* on the pelvic region of primates has practical and ethical constraints that have largely precluded *in vivo* approaches such as XROMM or cineradiography (but see [28,91]). Our simulated measures of hip abduction were previously compared with passive range of motion data from anaesthetized primates [2,7,35], with the *in silico* data consistently approaching the live animal values ([3]; see also electronic supplementary material S1). Although this validation with *in vivo* data is reassuring, the *in silico* models only reconstructed hip abduction in a single position and did not account for overlying tissues (e.g. cartilage, muscles and skin; see [92]) or all possible movements of the femur (see [37,38]). It is unknown, for example, how sensitive *abduction angle* is to slight changes in the positioning of the hip joint centre or out-of-plane joint rotations and/or translations. Our method for reconstructing the range of abduction may be applicable to other vertebrates but should be cautiously applied, especially to taxa with proximal femora that may promote more dynamic hip joint centre locations (e.g. saddle conformation in birds [93] and ovoid joint surface running onto femoral neck in lizards and crocodiles [94]).

Overall, the SEM methodology presented here demonstrates a fruitful path for future palaeobiological and biomechanical studies. All terrestrial vertebrates are governed by similar musculoskeletal contraints that simultaneously facilitate and limit locomotion depending on ecological context [95,96]. While our pathways to performance may contain some of the same causal links across groups, the relative strength of linkages between morphology and performance often differ substantially. Future applications of our systems-based approach may allow researchers to identify how to infer aspects of a species' palaeobiology based on fragmentary or isolated fossils. For example, the path models here show that *NSA* strongly tracks hip abduction across all extant anthropoids. By inference, the high *NSA*s observed in early fossil hominoids (approx. 135°; *Morotopithecus bishopii* [97], *Ekembo nyanzae* [32]) suggests that these animals would have had expansive hip abduction capabilities, typically associated with arboreal versatility in modern primates. This would be significant in the context of hominoid evolution because many early hominoids are interpreted as having more ‘monkey-like’ postcranial morphology that is inconsistent with the orthograde, forelimb-dominated behaviours characterizing all living hominoids [1]. While we only used directly measured variables, SEM can also be used to assess how unobserved, or latent, predictor variables influence outcomes. This may occur when a variable is quite abstract (e.g. animal motivation in sprint speed [98]) or a composite of multiple linear measurements. Future studies may employ latent variables when a hypothetical predictor variable probably influences a system, but the specific mechanism or trait is not directly measurable. Lastly, SEM may be useful for studies of biomechanical systems more broadly, especially through innovative approaches (i.e. musculoskeletal and kinematic modelling (e.g. [15,20]), musculoskeletal mapping (e.g. [99,100]), XROMM (e.g. [101])) where both body morphology and functional or performance data can be captured in a single individual.

## 5. Conclusion

This study demonstrates the effectiveness and potential of SEM—particularly regularized SEM—in anthropology, biomechanics and functional morphology. Our path models elucidate biologically important relationships between morphology and performance in extant primates. Through quantification of the relative strengths of multiple variables simultaneously and standardized variable selection techniques, regularized SEM shows what matters (and what does not) in a biological system. Key morphological traits, even from a single bone, can be informative about locomotor capabilities, which may have broad applications for reconstructing primate locomotor evolution and palaeoecology. The application of SEM to palaeobiological questions should be expanded to the study of structure–function relationships in other vertebrates, ideally incorporating live animal morphology and performance data when possible.

## Data Availability

The datasets and code supporting this article have been uploaded as part of the supplementary material [102].
